# Matching-range-constrained real-time loop closure detection with CNNs features

**DOI:** 10.1186/s40638-016-0047-x

**Published:** 2016-09-19

**Authors:** Dongdong Bai, Chaoqun Wang, Bo Zhang, Xiaodong Yi, Yuhua Tang

**Affiliations:** 1 College of Computer, National University of Defense Technology, Changsha, China; 2 State Key Laboratory of High Performance Computing, National University of Defense Technology, Changsha, China

**Keywords:** Loop closure detection, CNNs, Feature compression

## Abstract

The loop closure detection (LCD) is an essential part of visual simultaneous localization and mapping systems (SLAM). LCD is capable of identifying and compensating the accumulation drift of localization algorithms to produce an consistent map if the loops are checked correctly. Deep convolutional neural networks (CNNs) have outperformed state-of-the-art solutions that use traditional hand-crafted features in many computer vision and pattern recognition applications. After the great success of CNNs, there has been much interest in applying CNNs features to robotic fields such as visual LCD. Some researchers focus on using a pre-trained CNNs model as a method of generating an image representation appropriate for visual loop closure detection in SLAM. However, there are many fundamental differences and challenges involved in character between simple computer vision applications and robotic applications. Firstly, the adjacent images in the dataset of loop closure detection might have more resemblance than the images that form the loop closure. Secondly, real-time performance is one of the most critical demands for robots. In this paper, we focus on making use of the feature generated by CNNs layers to implement LCD in real environment. In order to address the above challenges, we explicitly provide a value to limit the matching range of images to solve the first problem; meanwhile we get better results than state-of-the-art methods and improve the real-time performance using an efficient feature compression method.

## Background

A simultaneous localization and mapping systems (SLAM) algorithm aims to map an unknown environment while simultaneously localizing the robot. Loop closure detection (LCD) is the technique of determining whether a mobile robot is back to a previously visited location, and it is critical for building a consistent map of the environment by correcting the localization errors that accumulate over time. Therefore, LCD is considered one of the most essential techniques in SLAM. To develop a LCD algorithm, one class of popular and successful techniques is based on matching the current view of the robot with those corresponding to previously visited locations in the robot map. In this case, LCD becomes an image matching problem, which typically includes two steps, image description and similarity measurement.

The state-of-the-art algorithms take advantage of the bag-of-words (BoW) model [1–3] to describe images. The BoW model clusters the visual feature descriptors in images, builds the dictionary, and then finds the corresponding words of each image. The BoW model is commonly used in visual features like SIFT [4], Surf [5] which have achieved great success in the past years. Despite significant progress in visual LCD, challenges still remain especially in dynamical and large-scale environment. As robots aim at long-term autonomous operations in the long period of time, such as days, weeks, or months, they are faced with environment that can undergo dramatic condition change and viewpoint change over time. Unfortunately, the hand-craft methods can not deal with these situations very well. Recent progress in the computer vision and machine learning community has shown that the features generated by convolutional neural networks (CNNs) outperform other methods in a variety of visual recognition, classification, and detection applications [6]. CNNs have been demonstrated to be versatile and transferable, that is to say, even though they were trained on a very specific target task, they can be successfully used for solving different problems and may outperform traditional hand-craft features [7, 8].

However, when we actually use these features generated by CNNs layers in a practical environment, two challenges appear. Firstly, the adjacent images in the dataset of LCD might have more resemblance than the images that really form the loop closure, so the algorithm tends to identify the adjacent images as loop closure, which is certainly not preferred. Secondly, the feature matching is computationally intensive because the dimension of features generated by CNNs may be very large, and LCD may have to compare the current image to a large amount of pre-captured images in order to decide whether the robot returns to previously visited positions. This can not satisfy strong request for real-time performance in robotic applications.

Against the background, in this paper we provide two solutions to address the above two challenges. Firstly, we explicitly provide matching range of candidate images to prevent images matching with their adjacent images. Meanwhile, we get better performance than state-of-the-art algorithms by adapting the matching range. Secondly, we provide a efficient feature compression method to reduce the dimension of feature generated by CNNs layers, which boosts real-time performance with marginal performance loss.

The rest of this paper is organized as follows. “Related work” section gives a brief introduction to the related work on LCD, CNNs, and datasets used in our subsequent experiments. In “Matching-range-constrained visual loop closure detection” section we present the details of Places CNNs model and how it is used to generate image descriptors. “Real-time large-scale visual loop closure detection” section shows algorithm and experiment results on the compression algorithm to realize real-time LCD. Finally, we conclude the paper in “Conclusions” section with a short discussion and future work.

## Related work

### Loop closure detection

The focus of research in LCD has recently moved from recognizing previously visited place without significant appearance changes [9, 10] to more realistic dynamical environment. Methods that address the LCD problem span from matching sequences of images [11, 12], transforming images to becoming invariant against common scene changes such as shadows [13, 14], learning how environments change over time and predicting these changes in image space [15−17], building up LCD hypotheses over time [18, 19], and building a map of experiences that cover the different appearances of a place over time [20].

### Deep convolutional neural network based feature

Previous works mostly relied on the hand-crafted traditional features or operated on the raw pixel levels [21]. These hand-crafted features are designed by experts having a lot of domain-specific knowledge. However, robot may be faced with a variety of complex and changeable environments during the process of localization. So it is very challenging for any people to take all factors affecting the performance of visual LCD into consideration.

Recently, there has been a trend in exploiting features generated by CNNs in computer vision, especially in the field of object recognition and detection [6]. A comprehensive evaluation further demonstrates the advantages of deep CNNs features with respect to shallow hand-crafted feature for image classification [22]. The advantage is that researchers will be free from mastering the knowledge of specific domains and the CNNs architecture can be used for many different domains, especially in visual systems with minor changes. CNN is a well-known architecture proposed by LeCun et al. [23] to recognize hand-written digits. Several research groups have recently shown that CNNs outperform classical approaches for object classification or detection that are based on hand-crafted features [8, 24]. The open-source software Caffe [25] provides pre-trained CNNs architectures for a variety of recognition tasks, which greatly reduces the difficulty in deploying and training CNNs for different tasks.

Hou et al. [7] were the pioneers to consider using features generated by CNNs layers for visual LCD. They use a public pre-trained CNNs model, Places CNNs, trained on the scene-centric dataset Places [26] with over 2.5 million images of 205 scene categories, as an efficient whole-image descriptor generator for LCD. They comprehensively compared the performance of Places CNNs model’s all layers by using the euclidean distance as the similarity measurement. Their work demonstrated that the pool5 layer provides the best image descriptors in terms of both detection accuracy and dimension of feature among all Places CNNs descriptors.

### Dataset

Experiments are conducted on two publicly available datasets with known frame correspondences, City Centre and New College built by Cummins et al. [9], firstly used by their loop closure detection algorithm called FAB-MAP. These two datasets are viewpoint change datasets widely used in visual SLAM research and in LCD in particular. The two datasets contain 2474 and 2146 images, respectively. Images are numbered sequentially in the order of collection. The camera was mounted on a pan-tilt and collects images from the left and right of the robot. Image collection was triggered every 1.5 m (on the basis of odometry) by the robot when it is driven through an outdoor urban environment with stable lighting conditions. The vehicle is in motion while the images are collected, so the robot travels same distance between the collection of the right and left images. Obviously, these two datasets exhibit strong viewpoint change. Ground truths in terms of true loop closures are also available. Details of these two datasets are available online.^1^

## Matching-range-constrained visual loop closure detection

In this section, we first explain the reason for setting the matching range and choose the matching range by evaluating the precision–recall performance.

### Image descriptor

In our experiment, we use the Places CNNs model trained on a scene-centric database [26] and constructed by Caffe [25]. The architecture of this pre-trained CNNs model is briefly summarized in Table 1. This CNNs model is a multi-layer neural network that mainly consists of three types of layers: five convolutional layers, three max-pooling layers and three fully connected layers. Note that a max-pooling layer only follows the first, second, and fifth convolutional layer but not the third and fourth convolutional layers.

**Table 1:** Architecture of the Places CNNs model and the dimension of the feature of each layer

	Convolutional								Fully connected		
Layer	CONV1	POOL1	CONV2	POOL2	CONV3	CONV4	CONV5	POOL5	FC6	FC7	FC8
Dimension	290,400	69,984	186,624	43,264	64,896	64,896	43,264	9216	4096	4096	1000

### Image matching

By using pre-trained CNNs model, we can create an image descriptor from each layer in the CNNs. That is, when we provide an image to CNNs, the output of each layer of CNNs is considered as a feature vector *u* of the image. In addition, we normalize these feature vectors to be unit vector *U*. We adapt the precision−recall curve as the performance evaluation criteria and euclidean distance for similarity measurement. The precision−recall curve is a standard evaluation method widely used in pattern recognition and in LCD particularly. To produce the precision−recall curve of a given image descriptor from CNNs layers, we compute feature vector *U* of the current view of robot and then find its nearest neighbor in the robot map that corresponds to previously visited locations according to the euclidean distance. Then we set a threshold on the euclidean distance to determine whether loop closure can be accepted, and we get precision and recall pairs by comparing our results with the ground truth of the dataset after all images in the dataset are considered. Finally, we can produce the precision−recall curve by varying the value of the threshold.

### Matching-range setting

**Fig. 1:**
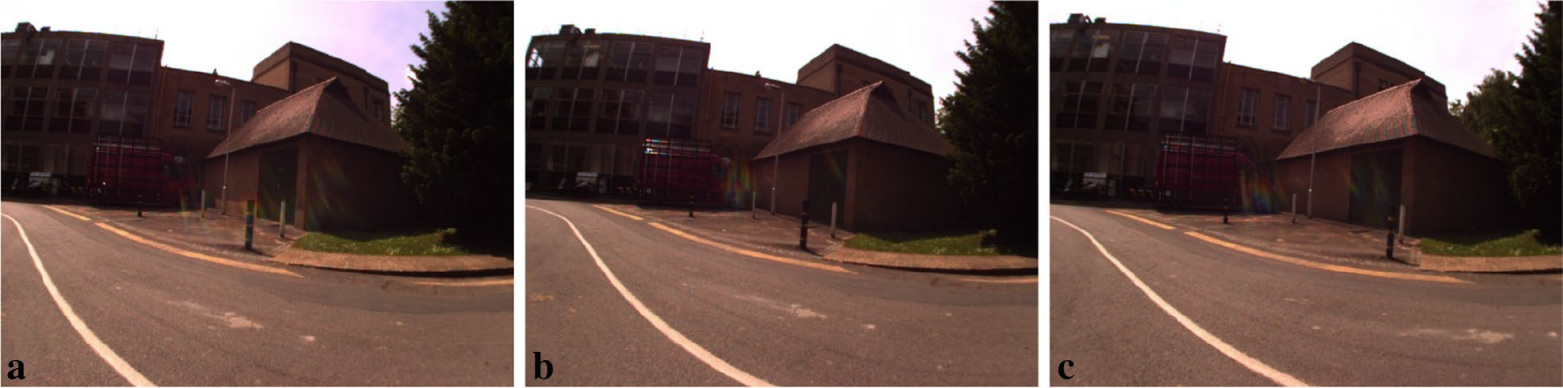
Three example images of City Centre dataset. **a** NO.0349, **b** NO.1399, **c** NO.1401

The feature provided by pool5 is an efficient whole-image descriptor in the application of visual LCD, the robot collects one image every 1.5 m, so the pool5 may generate more resemblance descriptors for adjacent images than the images forming actual loop closure by the euclidean distance measurement. For example, the distance between the 1399th and 1401th image is 0.2342 and the distance between 349th and 1401th is 0.3302; obviously the 1399th image is more similar to 1401th image than the 349th image. The algorithm without setting the matching range would believe the 1399th and 1401th image form the loop closure (these images are all chosen from City Centre and are shown in Fig. 1). But the distance between the collection of the 1399th image and the 1401th image is only 3 m, due to the robot collected images every 1.5 m, while the ground truths reveal the image really matching the 1401th image is 349th image. In this situation, the algorithm will get a number of errors and greatly degrade its performance.

**Fig. 2:**
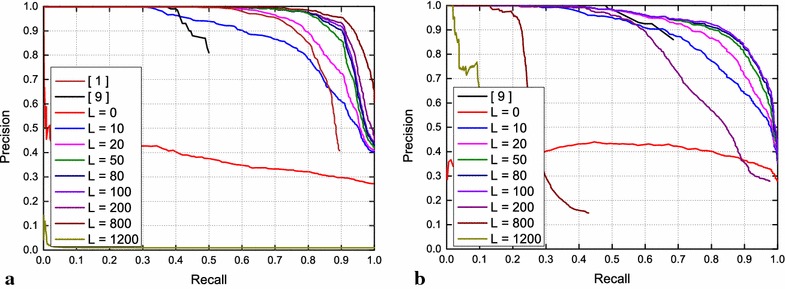
Visual loop closure detection precision−recalls on City Centre dataset and New College dataset use feature generated by pool5 with different value *L*. **a** Precision−recalls on City Center dataset, **b** precision−recalls on New College dataset

In order to deal with this problem, we provide a constraint to limit the matching range of images for the current position to determine the loop closure image. Concretely, if the current image number is *N* and the number of excluded images is *L*, the algorithm will only determine whether loop closure appears only from the image number 1 to image number *N* − *L*. If the range is set properly, the distance (on the basis of odometry) between the image *N* − *L* and image *N* is long enough so that the difference of descriptors generated by pool5 between the image *N* and the image *N* − *L* is distinguishable for the LCD algorithm. In this case, the above problems can be properly addressed. The precision−recall curve for different matching ranges on the City Centre and New College dataset is shown in Fig. 2 given different *L* values. Specifically, the *L* = 0 curve represents the case where the current image is compared with all previous images, and the resulted precision−recall performance is very poor due to high resemblance with the adjacent images, which do not form loop closures.

For the sake of evaluating the effects of matching ranges on LCD performance, we implement the algorithm with different *L* values on the City Centre and New College dataset. Figure 2 shows the resulting precision−recall curves for various experimental settings. The experiments show that performance improves as the *L* value increases at the beginning and then degrades drastically as shown in *L* = 1200 in Fig. 2a. The reason of this phenomenon is that, at first, the increase in the number of excluded images can reduce likelihood of occurrence of above problem; however, if the algorithm excludes too many images will result in a small matching range for an image, which may lead to the case that no images in the matching range can really form loop closure with current image revealed by the ground truth, but the LCD algorithm will still identify a “wrong” image for the current image to form loop closure based on euclidean distance.

As shown in Fig. 2a, in the City Center dataset, *L* = 800 achieves the best precision−recall performance. If the precision is guaranteed to be 100 %, the achievable recall is approximate 10 % better than the benchmark proposed in [7] and 35 % better than the benchmark FAB-MAP proposed in [9]. Figure 2b illustrates the effects of selected *L* values on the performance in the New College dataset, where the *L* = 100 is optimal. If the precision is guaranteed to be 100 %, the algorithm achieves comparable recall performance with FAB-MAP [9]. Therefore, the optimal searching range settings represented by *L* depends on the dataset, which reflects the explored environments and the chosen routing of the robots. Hence, we are investigating the methodology of training *L* via on-line observations, which is left for our future works.

## Real-time large-scale visual loop closure detection

The results have demonstrated that by setting proper matching ranges, the features from layer pool5 is robust against viewpoint change on City Centre and New College dataset. However, computing the euclidean distance between many 9216 dimension pool5 feature vectors may become a computationally intensive operation and a bottleneck of the real-time performance. Therefore, directly using the features extracted by pool5 may not satisfy real-time demand of robotic application, especially in large-scale scenes. Hence, the high-dimensional CNNs features may be compressed as in [27]. In this section, we explore the power of locality-sensitive hash (LSH) functions to address the challenge. The results demonstrate that speed-ups of four to seven times can be achieved with negligible performance degradation in terms of the precision−recall metric.

### Feature compression

As the dimension of feature extracted by pool5 is very large, we naturally think of compressing the feature to low-dimensional vectors to accelerate LCD algorithm. We adopt the LSH method proposed by Charikar [28], which uses random hyperplanes to generate an LSH function. This algorithm can preserve the cosine similarity between vectors and shapely reduces the dimension of vectors, which in turns greatly reduces the time of calculating our feature distance similarity matrix.

However, the compression may be achieved at cost of performance degradation on detection accuracy. Therefore, we generate compression features for all images in City Centre and New College dataset with various compression ratios to find the trade-off between real-time and precision−recall performance.

### Image matching

The benchmark we adopted is non-compressed cosine similarity, which calculates the normalized inner product of two original features expressed as

1 $$\cos (\theta (u,v))=\frac{|u\cdot\,v|}{\sqrt{|u||v|}}, $$

where $\theta (u,v)$ is the angle between the vectors *u* and *v*. $|u\cdot\,v|$ is the inner product of *u* and *v*, and |*u*| and |*v*| represent the length of vectors *u* and *v*, respectively. Following the random hyperplanes strategy in [29], the LSH proposes to use a collection of random vectors in a *k*-dimensional vector space. We first generate a spherically symmetric random vector *r* of unit length from this *k*-dimensional space. We then define a hash function, $h_{r}$, as:

2 $$h_{r}(u)=\left\{ \begin{array}{ll} 1 &\quad r\cdot\,u\ge 0\\ 0 &\quad r\cdot\,u<0\\ \end{array}\right.,$$

For vectors *u* and *v*, we have

3 $$ Pr[h_{r}(u)=h_{r}(v)]=1-\frac{\theta (u,v)}{\pi }, $$

which is proved by Goemans and Williamson [30] and reveals that the probability of a random hyperplane separating two vectors is proportional to the angle between the two vectors (i.e., $\theta (u,v)$). From Eq. (3) we may infer that

4 $$ \cos (\theta (u,v))=\cos ((1-Pr[h_{r}(u)=h_{r}(v)])\pi ), $$

According to Eq. (4), we can get $Pr[h_{r}(u)=h_{r}(v)]$ by the calculation of hamming distance^2^ between the vectors *u* and *v*. It is noted that the cosine similarity measurement is also equivalent to the euclidean distance measurement for normalized vectors. Hence, the hamming distance measurement is equivalent to euclidean distance measurement used in our previous experiments. In addition, the computation of hamming distance between two bit vectors has many advantages in comparison with euclidean distance, such as time and memory saving. Intuitively, Eq. (4) is stochastic, and based on numerical evaluation, we should generate sufficient random hyperplane to achieve more satisfactory approximation. As we generate a larger number *d* of random vectors, the hamming distance may estimate the euclidean distance between two vectors more accurately, however, at the cost of increasing the amount of computation. Hence, we should choose a proper *d* to strike a beneficial trade-off between the approximation accuracy and the computation complexity.

### Algorithm implementation

In the previous subsection, we introduced the algorithm for feature compression. The complete algorithm for the LCD is as follows:

1. Firstly, we produce *n* feature vectors generated by CNNs layer of pool5 (for City Centre and New College dataset, n is equal to 2474 and 2146, respectively) using Caffe [25].
2. Secondly, we generate *d*$(d<k)$ unit random vectors $\{r_{0},r_{1},\ldots ,r_{d}\}$. Each $r_{i}$ has *k* elements (for feature extracted by pool5, *k* = 9216), and each elements is sampled from a Gaussian function with mean 0 and variance 1. We then put the *d* vector $\{r_{0},r_{1},\ldots ,r_{d}\}$ together into a matrix *D* of dimension $k\times d$.
3. Thirdly, we produce the inner product between D and every feature vector *v* with dimension of *k* to get vector $u=D^{T}v$. Then, for every vector *u*, we use the function $h_{r}(u)$ [as Eq. (2)] to produce compressed feature $\bar{u}$ as: $\bar{u}=\{h_{r1}(u),h_{r2}(u),\ldots ,h_{rd}(u)\}$. Hence, each compressed feature is represented by a bit stream of length *d*. The time complexity of steps 2 and 3 is *O*(*nkd*) time.
4. For every image in the City Centre and New College dataset, we produce its compression feature vector $\bar{u}$ and then find its nearest neighbor from the previous images in the dataset by calculating the hamming distance of their compressed features. The time complexity of this step is $O(n^{2}d)$.
5. Overall, the time complexity of the total algorithm is $O(nkd+n^{2}d)$ to calculate the full similarity matrix. However, the time complexity of using the cosine similarity to produce the full similarity matrix would be $O(n^{2}k)$. For large-scale LCD, *n* must be very large, so we can get great speed-up factor from the algorithm. Further more, with the increase in *n*, the algorithm will achieve greater speed-up factor.

### Evaluation

**Fig. 3:**
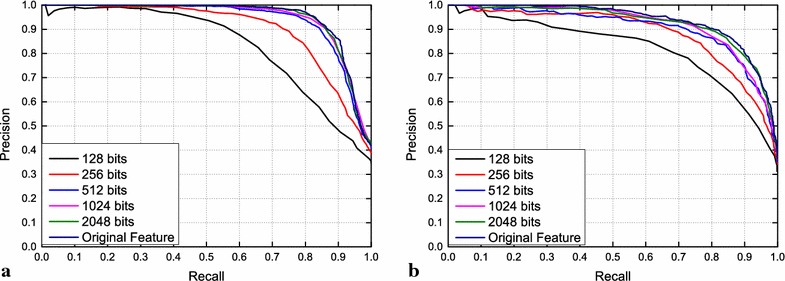
Hamming distance over the original feature vectors of 9126 generated by pool5 can be closely approximated by the hamming distance over bit vectors of length 1024 with marginal precision loss. **a** City Centre dataset, **b** New College dataset

In this paper, we implement the algorithm and compare the visual LCD performance achieved with the compressed feature vectors of pool5 of different lengths *d* = (128, 256, 512, 1024, 2048) bits on the City Centre and New College datasets in Fig. 3. Since the hamming distance over bit vectors is more computationally efficient, the best-matching image among 2474 candidates can be found within 5.67 ms on a standard desktop machine with a 3.60-GHz CPU with four cores and 8GB memory. This corresponds to a speed-up factor of ~4 compared to the algorithm that uses the euclidean distance over the original pool5 features, which required 21.3476 ms for each candidate. Calculating the hashes requires 1.0913ms using a non-optimized Python implementation. Table 2 summarizes the required time for the main algorithmic steps. We can see that the LSH enables real-time visual LCD using CNNs-based features on large-scale places.

**Table 2:** Runtime comparison between original features and compressed features

	Original pool5	128 bits	256 bits	512 bits	1024 bits	2048 bits
Feature compression	–	0.1492 ms	0.2822 ms	0.5493 ms	1.0913 ms	2.1550 ms
Match 1000 candidates	21.3476 s	3.3334 s	3.4750 s	3.8738 s	4.5803 s	5.8495 s

## Conclusions

Our paper presented a thorough investigation on the effects of matching range for CNNs-based LCD. By proper selecting the matching range, we get better performance than the state-of-the-art algorithms on the City Centre and New College dataset. In addition, we provide acceleration solutions for large-scale real-time LCD by using a specialized LSH method to compress high-dimensional CNNs features.

Note that our study is still preliminary at this point since the matching range is dataset-sensitive and chosen manually in this paper. In our future works, we will employ machine learning algorithm to enable the LCD algorithm autonomously finding the optimal matching ranges for various datasets. Also, we may use the point location in equal balls algorithm [31] to accelerate the process of finding most similar images for current viewpoint. Also, we plan to train CNNs specifically for the task of LCD under dynamic environment to get a better descriptor.

## Footnotes

1 http://www.robots.ox.ac.uk/~mobile/IJRR_2008_Dataset/.

2 Hamming distance is the number of bits which differ between two binary strings. Thus, the hamming distance between two strings A and B is $\sum _{i}|A_{i}-B_{i}|$.
